# Distribution pattern of large old *Ginkgo biloba* in China under climate change scenarios

**DOI:** 10.1002/ece3.11367

**Published:** 2024-05-16

**Authors:** Chunping Xie, Chang Liu, Houhe Wang, Dawei Liu, Chi Yung Jim

**Affiliations:** ^1^ Tropical Biodiversity and Bioresource Utilization Laboratory Qiongtai Normal University Haikou China; ^2^ Nanjing Institute for Comprehensive Utilization of Wild Plants, China Co‐ops Nanjing China; ^3^ Faculty of Criminal Science & Technology Nanjing Police University Nanjing China; ^4^ Department of Social Sciences and Policy Studies Education University of Hong Kong Tai Po, Hong Kong China

**Keywords:** BIOCLIM, DOMAIN, climate change, distribution pattern, *Ginkgo biloba*, large old tree

## Abstract

Large old *Ginkgo biloba* trees (LOGTs), with profound ecological and cultural significance in China, face increasing threats from climate change and human activities. We employed the BIOCLIM and DOMAIN species distribution models to predict their spatial patterns under the present climate and doubled‐CO_2_ climate change scenario in 2100. We collected 604 validated LOGT occurrence records and data on 19 bioclimate factors for the analysis. Our study yielded a LOGT geographic distribution pattern covering a wide latitudinal belt extending from south subtropical to temperate zones in central and eastern China, concentrating in low elevations and coastal regions. The principal component analysis identified the dominant bioclimatic factors shaping their distribution, namely annual precipitation and low winter temperatures. BIOCLIM and DOMAIN generated predicted suitable habitats that match the present distribution range well. However, under the future climate scenario, the models indicated habitat retentions mainly in the core distribution areas and habitat losses mainly in the southern edge of the present range and scattered pockets elsewhere. Some retained habitats, including excellent ones, will suffer from fragmentation. The predicted new habitats may permit some range expansion and migration but are beset by small patch size and large interpatch distance, bringing fragmentation and gene flow restrictions. The anticipated projected range decline highlights considerable threats climate change poses to the long‐term survival of the precious natural‐cum‐cultural resource. Understanding the distribution patterns and underlying drivers and distillation of practical conservation measures can foster sustainable management vis‐a‐vis the looming global change.

## Graphical abstract

The study investigates the distribution of large old *Ginkgo biloba* trees (LOGTs) in China and their vulnerability to climate change and human activities. Using species distribution models, the study predicts the spatial patterns of LOGTs under present and future climate scenarios. The results reveal a wide latitudinal distribution belt for LOGTs, with concentration in low elevations and coastal regions. While the models indicate retained habitats in core distribution areas under future climate scenarios, there are predicted habitat losses in the southern edge of the range and scattered pockets elsewhere, highlighting the threats climate change poses to the long‐term survival of these valuable natural and cultural resources.

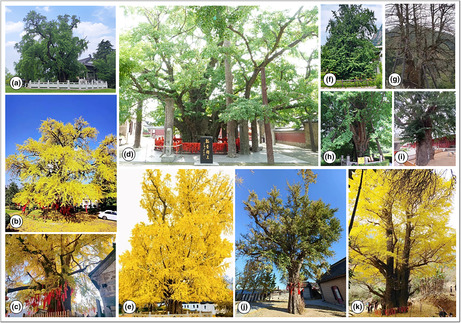

## 1 INTRODUCTION

As an indispensable part of natural ecosystems, large old trees (LOTs) harbor profound ecological values and cultural significance (Blicharska & Mikusiński, 2014). Many studies have demonstrated LOT's critical role in maintaining biodiversity, ecological balance, cultural heritage, and scientific research (Jim, 2017; Lindenmayer, 2017). They support a wide array of companion life forms such as lichen, moss, and fungus due to their structural complexity and sheer size, providing a diverse assortment of microhabitats to support many species, including rare and endangered ones (Kozák et al., 2023; Le Roux et al., 2014; Lindenmayer, 2017). In urban areas, preserving large trees can effectively conserve local biodiversity (Stagoll et al., 2012).

LOTs furnish a reliable record extending over centuries or even millennia of environmental changes, serving as living archives for scientific research (Piovesan & Biondi, 2021). LOT tree rings provide invaluable information for archeologists and paleoclimatologists to decipher past human activities, environmental changes, and climate shifts (Anchukaitis, 2017). In addition, LOTs embody the cultural heritage and identity of indigenous communities (Atindehou et al., 2022). The long‐living and transgenerational companions are deeply rooted in folklores, arts, and spiritual practices of many societies (Blicharska & Mikusiński, 2014; Huang et al., 2020). Protecting these treasured trees allows the preservation and transfer of traditional knowledge to future generations (Ulluwishewa et al., 2008). More research and conservation efforts are needed to understand and protect these monumental living legacies.

Climate change, coupled with human activities, has continued to endanger LOTs worldwide in recent decades (Li & Zhang, 2017; Lindenmayer et al., 2012). Several related factors threaten the survival of these venerable doyens that have often lived for centuries. Rising temperatures and intensifying droughts associated with climate change have weakened LOTs, rendering them more prone to pests, diseases, and wildfires (Lloret & Batllori, 2021). Aggravated moisture deficit has compromised LOT resilience, making them more susceptible to invasive pathogens and insects growing aggressively under warmer climates (Vacek et al., 2023). In addition, the increasingly hot and dry conditions raise the flammability of forests, and constituent veteran trees adapted to wet environments could be damaged or killed by fires (Lindenmayer & Laurance, 2017; Shuman et al., 2017).

Climate change may directly dampen the physiological and reproductive processes of LOTs. Temperature rise can induce heat stress to reduce their survival and growth. Some trees' ability to become LOTs may be compromised or arrested (Allen et al., 2010). Changes in precipitation patterns and water availability may disrupt the water balance of LOTs to bring drought stress or waterlogging (Mattos et al., 2023). These climate change consequences can alter the distribution and abundance of LOT habitats. Furthermore, climate change can indirectly exacerbate the effects of nonclimatic factors on LOTs. For example, the impacts of deforestation and land‐use changes, driven by human activities, can be aggravated by climate change (Dale, 1997). Rising temperatures and altered precipitation patterns can accelerate soil erosion, reduce soil fertility, and increase pest and disease outbreaks (Skendžić et al., 2021). Such collateral changes can further degrade the habitat quality of LOTs and limit their ability to persist and regenerate. By highlighting the importance of climate change assessments, this study improves understanding of the potential threats facing this iconic species. It emphasizes the need for proactive measures to ensure their long‐term survival in a changing climate regime.

To better understand and protect these irreplaceable and vulnerable resources, comprehensive LOT studies have been conducted worldwide to map distributions, assess threats, and develop conservation strategies (Le Roux et al., 2014; Lindenmayer & Laurance, 2017; Malliarou et al., 2023). Among various research methods, the species distribution model (SDM) has emerged as an essential tool with diverse applications in LOT studies and conservation planning (Xie et al., 2020, 2022). SDM utilizes tailor‐made computer algorithms to predict potentially suitable habitats based on known occurrence records and environmental variables (Abdelaal et al., 2019; Zhang et al., 2023). The method enables researchers to locate existing individuals, identify survival and regeneration areas, map potential habitats, steer field surveys, and delineate LOT protection zones (Benner et al., 2019).

Moreover, by incorporating future climate projections, SDMs can assess climate change impacts and risks for LOTs. For instance, in the Caspian Hyrcanian Mixed Forest ecoregion (a UNESCO World Heritage Site near the Caspian Sea shores in Iran and Azerbaijan), only 10% of the currently suitable habitats will retain their suitability for *Taxus baccata* (English yew) in 2070. However, with the high emission RCP 8.5 global warming scenario, no stable suitable habitats will be left (Alavi et al., 2019). Furthermore, SDMs help identify key environmental factors shaping LOT distributions. The findings can inform conservation efforts, such as the BIOCLIM modeling of spatial pattern drivers in Sichuan, China, guiding the protection of threatened trees (Xie et al., 2022). Overall, SDMs can provide indispensable data and reliable predictions to locate priority conservation areas, monitor population changes, and safeguard these irreplaceable living legacies. The technique has become important for evidence‐based research and protection of LOTs worldwide (Nolan et al., 2022).

*Ginkgo biloba* L. (hereinafter referred to as “*Ginkgo*”) flourished in the Paleozoic Era about 270 million years ago, when a sizeable population was distributed widely in the Northern Hemisphere (Hsieh, 1992). Unfortunately, the Quaternary Glaciation brought drastic environmental upheavals, causing a catastrophic extinction of *Ginkgo* in most parts of Europe, North America, and Asia (Liang & Hou, 1990). Nowadays, only a few wild and semi‐wild *Ginkgo* trees survive as localized remains in several tiny and isolated sites in China, such as Tianmushan Mountain in Zhejiang Province. The relict species is recognized as the oldest living fossil in the world (Cao, 2016; Hsieh, 1992). According to historical records, *Ginkgo* was cultivated in China in the Shang Dynasty over 3000 years ago, with a long history of horticultural use and survival under human care (Chi et al., 2020). The vicissitudes of many old *Ginkgo* trees in many settlements reflect the contemporaneous interplay of natural, historical, cultural, and social changes.

Large old *Ginkgo* trees (LOGTs), an irreplaceable and important natural resource, are extremely valuable historical and cultural heritage. Widely relished and respected by people in different countries, China has the most LOGTs globally (Gong et al., 2008). They have high economic, ornamental, ecological, cultural, and special scientific research values. LOGT studies in China have mainly focused on resource quantity (Liu et al., 2015), distribution (Cao et al., 2019; Chi et al., 2020; Hsieh, 1992; Liang & Hou, 1990), growth status and rejuvenation techniques (Li et al., 2016; Yan et al., 2021), and molecular biology (Guan et al., 2016). Some studies focus on the limiting factors of current LOGT distribution (Chi et al., 2020). However, changes in LOGT distribution patterns in China in response to climate change have remained unclear, signifying a knowledge gap that deserves to be investigated. As global climate warming intensifies, we hypothesize that the LOGT habitats will be fragmented, and the southern edge of its current range will recede mainly northward.

This study examined the distribution patterns of LOGTs in China under current climate and future climate change scenarios using two SDMs, BIOCLIM and DOMAIN. The objectives are to (1) identify and evaluate the significant bioclimatic factors influencing the present LOGT distribution, (2) predict suitable habitats for future LOGT growth based on their current tree distribution and bioclimatic data, and (3) forecast changes in suitable habitats for LOGTs under a climate change scenario. The findings could contribute to conserving LOGTs, selecting appropriate ex situ conservation sites, and managing the sustainable use of this precious resource.

## 2 MATERIALS AND METHODS

### 2.1 Data collecting

**FIGURE 1:**
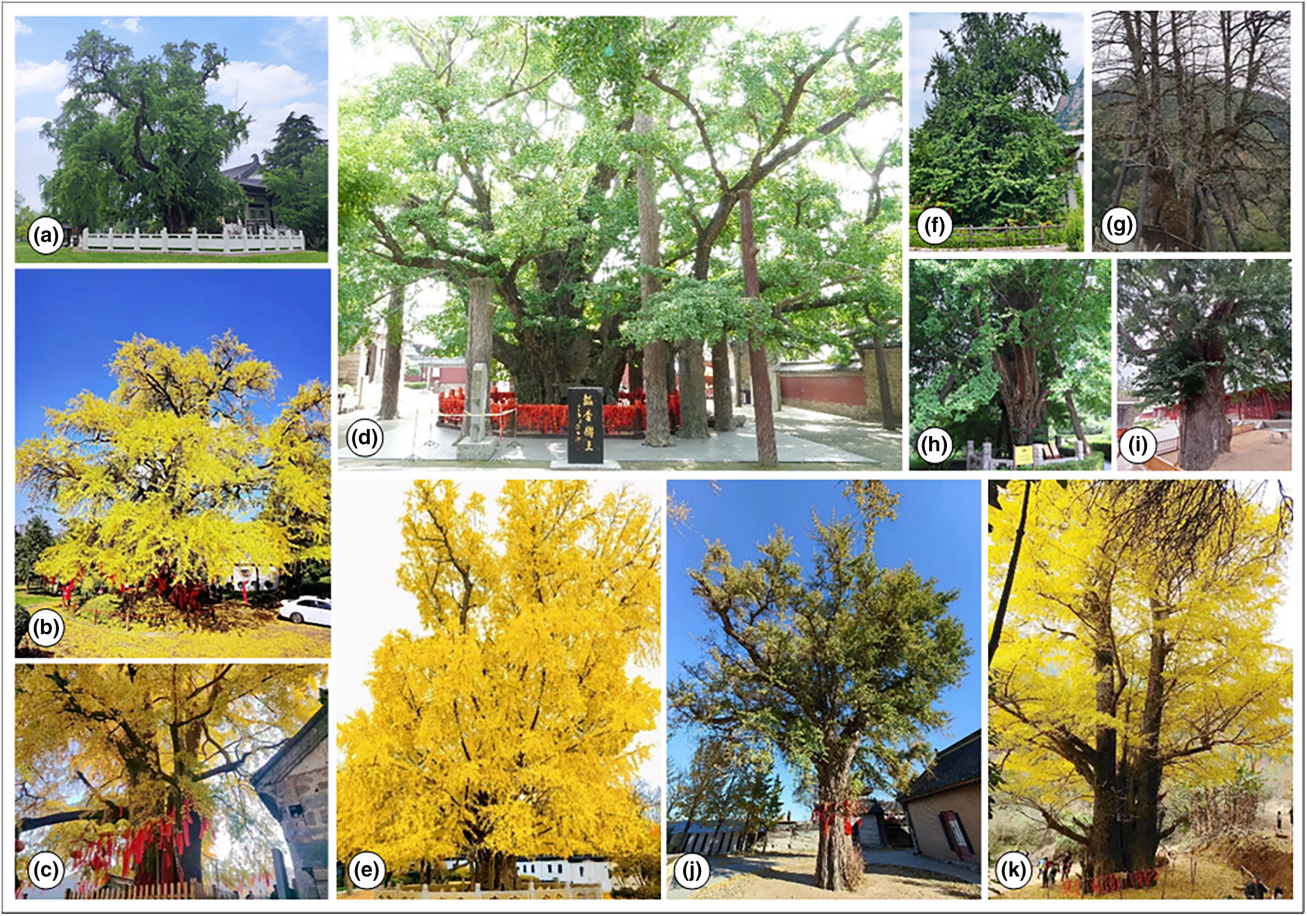
Notable samples of large old *Ginkgo* trees (LOGTs) in China. (a) Nanjing, Jiangsu (118°31′15.1″ E, 32°6′9.6″ N), estimated age about 1500 years; (b) Meitan, Guizhou (107°23′43.3″ E, 27°35′31.4″ N), 1400 years; (c) Weihui, Henan (113°59′40.9″ E, 35°41′22.5″ N), 1200 years; (d) Ju County, Shandong (118°44′36.9″ E, 35°36′6.8″ N), 4000 years; (e) Tongling, Anhui (118°5′47.8″ E, 30°55′9.1″ N), 1000 years; (f) Liuba, Shaanxi (106°59′22.2″ E, 33°43′20.1″ N), 4000 years; (g) Dong'an, Hunan (111°29′25.7″ E, 26°50′11.2″ N), 2500 years; (h) Tanze Temple, Beijing (116°2′14.5″ E, 39°54′42.8″ N), 1400 years; (i) Linyi, Shandong (118°20′44.1″ E, 35°4′41.3″ N), 900 years; (j) Dalian, Liaoning (121°24′3.8″ E, 39°0′23.2″ N), 1370 years; (k) Yudu, Jiangxi (115°19′35.2″ E, 25°45′37.2″ N), >1000 years. The authors measured the trees' geographic coordinates using a handheld GPS meter. Tree age was estimated by the local forestry department based on historical information or forest work experience.

**FIGURE 2:**
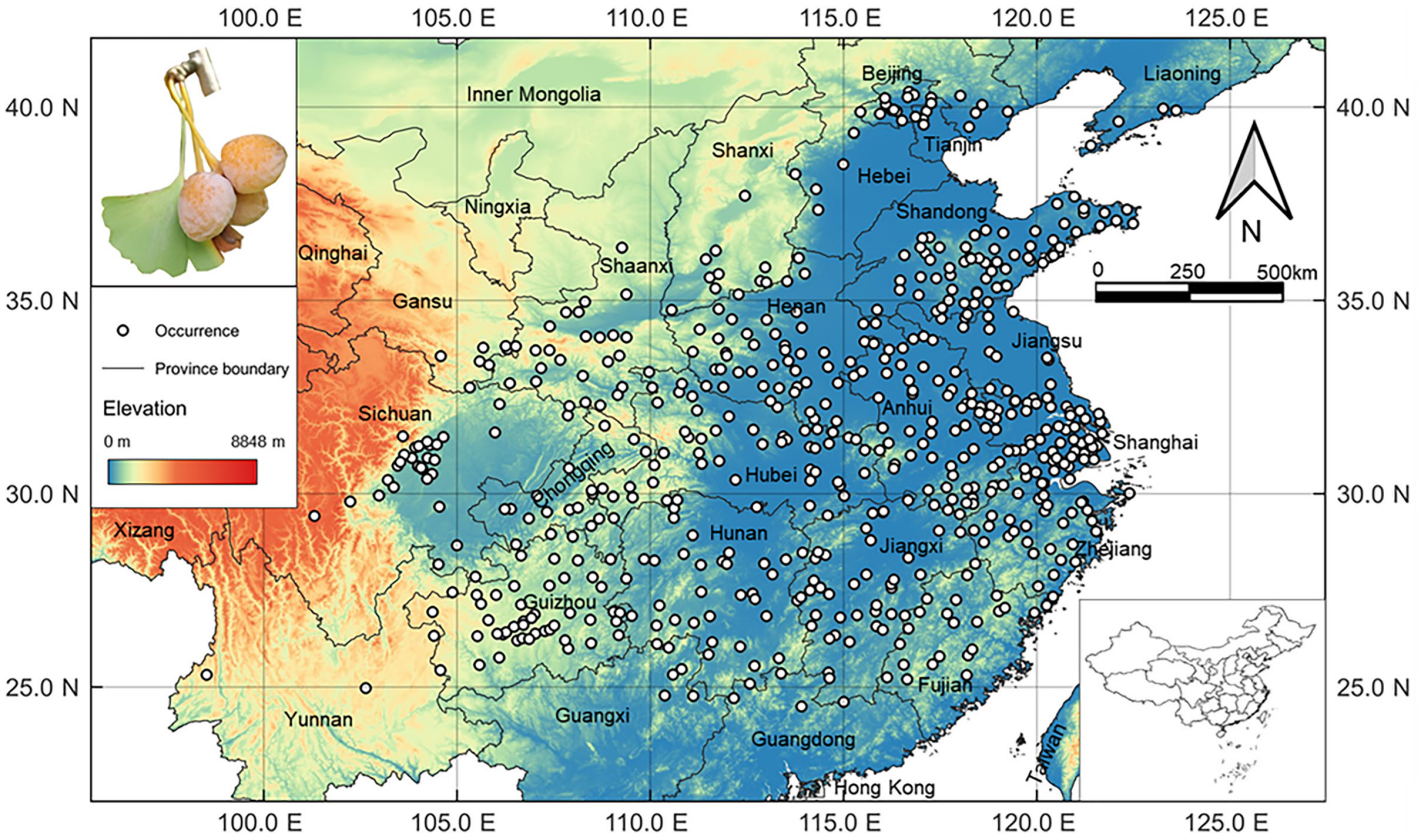
Locations of 604 present occurrence records (shown by white dots) of LOGTs in China. The inset image shows a leaf and fruits of *Ginkgo biloba*.

The geographical distribution data of LOGTs were collected from three sources. Based on *Technical regulation for surveying old and notable trees* published by the government's forestry department, LOGTs in this study were defined as over 100 years old (Lai et al., 2019). First, we gleaned 2225 records from the published literature on LOGTs. Second, investigations on LOGTs from 2010 to 2022 in 11 provinces and regions across East, Central, South, and Southwest China to collect 317 records (Figure 1). Third, we gathered 674 records from news reports related to LOGTs from 2015 to 2022 on major Chinese portal websites, such as Sina, 163, Sohu, CCTV, Ifeng, and Tencent. Only high‐quality data were used by deleting those without detailed geographical locations. In addition, Google Earth (http://earth.google.com) was used to check the locations and delete records with incorrect information. To reduce spatial autocorrelation and remove duplicate records, we scaled down to 604 sample locations at the county level for our study (Figure 2).

### 2.2 Environmental variables

This study used 19 bioclimatic variables to predict the potential distribution patterns of LOGTs at present and in 2100. The data were downloaded from the WorldClim website (http://worldclim.org) (Fick & Hijmans, 2017). The CCM3 model of the National Center for Atmospheric Research (Kiehl et al., 1998), which simulates the 2100 climatic scenario with a doubling of atmospheric carbon dioxide concentrations, was used for future climate projections. A spatial resolution of 2.5 arcminutes was used for present and future climate data sets. The environmental limiting factors for LOGT distribution in China were examined with principal component analysis (PCA) using the PAST 4.12b software (Hammer & Harper, 2001).

The 19 bioclimatic variables were beset by autocorrelation and multicollinearity. The highly correlated variables would introduce redundant information during model prediction to reduce modeling accuracy and reliability (Alavi et al., 2019). Using the usdm package in the R environment (Team, 2013), a procedure and variance inflation factor (VIF) were applied to choose only the variables with <5 VIF value (Abdelaal et al., 2019). Ultimately, six environmental variables, including bio6 (minimum temperature of the coldest month), bio8 (mean temperature of the wettest quarter), bio10 (mean temperature of the warmest quarter), bio11 (mean temperature of the coldest quarter), bio12 (annual precipitation), and bio13 (precipitation of the wettest month) were selected for modeling in Diva‐GIS 7.5 (Hijmans et al., 2001).

### 2.3 Model assessment

The selected LOGT data set was divided into two subsets. A random selection of 70% of the “presence points” was assigned to the training subset and the remaining 30% to the validation subset. We then sequentially loaded the randomly generated training subset files in DIVA‐GIS 7.5. Multiple distribution prediction layers were obtained by applying the previously generated bioclimatic variable data set for model computation. We averaged the multiple prediction values to obtain the potential distribution pattern of LOGTs in China based on BIOCLIM (Booth et al., 2014) and DOMAIN models (Carpenter et al., 1993). Subsequently, we imported the results into QGIS 3.22 for visualization processing (Moyroud & Portet, 2018).

Using the suitability index generated by BIOCLIM, the suitable habitats for LOGTs were classified into six levels: unsuitable (0%), low (0.0%–2.5%), medium (2.5%–5.0%), high (5.0%–10%), very high (10%–20.0%), and excellent (20.0%–41.0%) (Beaumont et al., 2005). The DOMAIN model's output values did not directly estimate suitability for LOGTs. Instead, they represent classification confidence. Human‐defined thresholds were necessary to determine suitable habitats based on DOMAIN model results (Sheng et al., 2022). The suggested thresholds for interpreting DOMAIN outputs, ranging from unsuitable to excellent, were −210 to 90, 90 to 92, 92 to 94, 94 to 96, 96 to 98, and 98 to 100, respectively.

Both models' performances were categorized as fail (0.5–0.6), bad (0.6–0.7), fair (0.7–0.8), good (0.8–0.9), and excellent (0.9–1.0) based on the test data's area under the curve (AUC) for receiver operating values (Abdelaal et al., 2019; Beaumont et al., 2005; Nolan et al., 2022).

## 3 RESULTS

### 3.1 Geographic distribution pattern

The spatial distribution of LOGTs in China covered a range demarcated approximately by 98.5° E–123.6°′ E and 24.5° N–40.4° N, as shown in Figure 2. Regarding the locations of the 604 valid occurrences by province, Shandong province had the highest frequency (68), followed by Anhui (57), Henan (49), and Jiangsu (47). Hubei, Guizhou, Zhejiang, Jiangxi, Hunan, and Sichuan had a medium frequency of 30–45. Shaanxi, Fujian, Chongqing, Guangxi, and Tianjin had a relatively low frequency of <30. The southernmost distribution occurred in Wongyuan (Guangdong), the northernmost in Huairou (Beijing), the easternmost in Dandong (Liaoning), and the westernmost in Tengchong (Yunnan). In addition, almost no LOGT records were found south of the Nanling Mountains. Therefore, the spatial distribution of LOGTs mainly covered the northern subtropical and temperate zones in central and eastern China. Areas that are too hot, cold, or dry had scanty or no LOGTs.

The altitudinal distribution of LOGTs extended broadly from low elevations to above 3000 m (Figure 2). The highest occurrence occurred in Ganzi (>3000 m) in Sichuan, while the lowest occurrence was in Sheyang (0 m) in Jiangsu. We divided the data into four altitudinal classes. The occurrences at low (<800 m), mid (800–1600 m), mid‐high (1600–2400 m), and high (>2400 m) elevations accounted for 83.6%, 14.4%, 1.8%, and 0.2% of the valid records, respectively. Notably, more than 80% of the occurrences were concentrated at low altitudes. The vertical distribution pattern suggests that LOGTs prefer low‐elevation habitats.

### 3.2 Limiting bioclimatic factors

**FIGURE 3:**
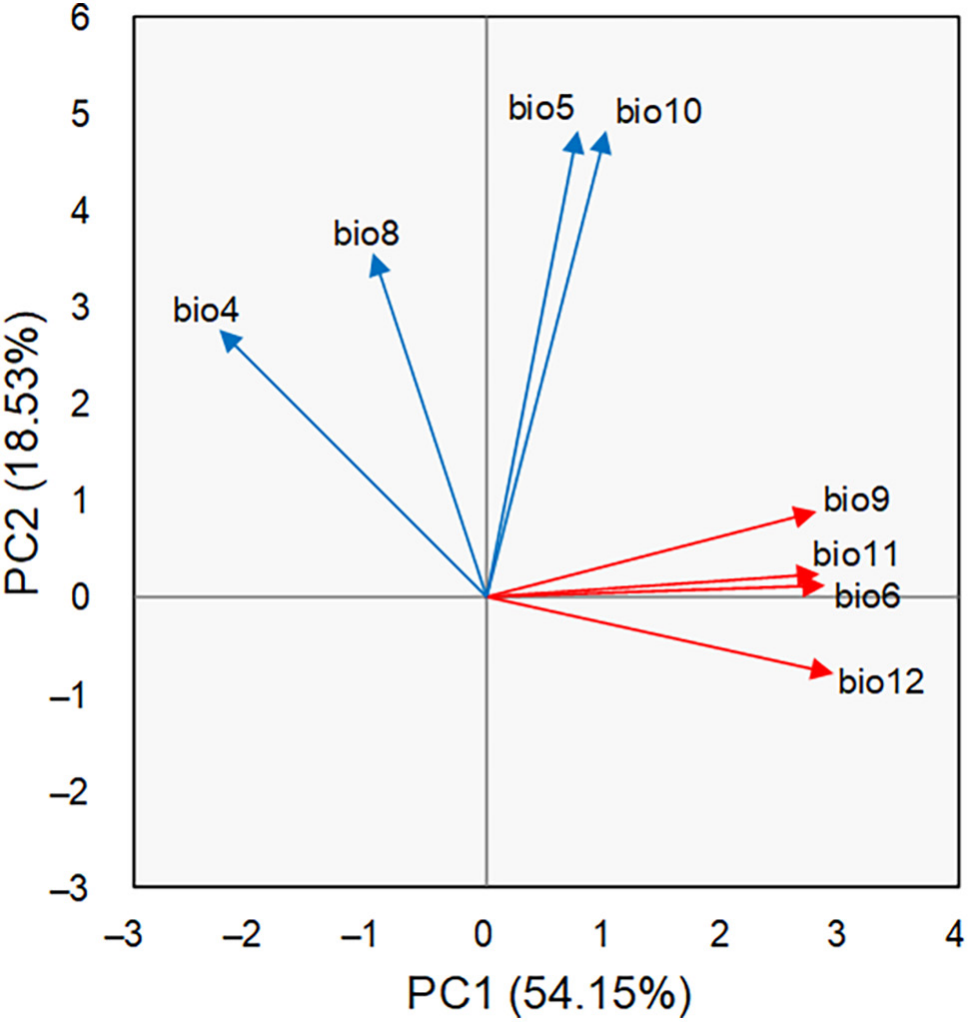
Principal component analysis (PCA) of 8 bioclimatic variables for the 604 occurrence records of LOGTs. The first PCA axis accounts for 54.15% of the total variance, and the second PCA axis accounts for 18.53%. The red and blue arrows represent the top four factors on the first and second PCA axes, respectively.

The PCA of independent bioclimatic variables reduced the dimensionality of the environmental space to identify the essential drivers of LOGTs distribution (Figure 3). The first and second principal components (PC1 and PC2) explained 54.15% and 18.53% of the variations, respectively, with a cumulative contribution exceeding 70%. Additionally, the eigenvalues of PC1 and PC2 were greater than 1 (10.29 and 3.52), indicating that they could represent the dominant environmental influence on LOGT distribution. In PC1 (red arrows), the top four factors were annual precipitation (bio12, 0.292), minimum temperature of the coldest month (bio6, 0.285), mean temperature of the coldest quarter (bio11, 0.281), and mean temperature of the driest quarter (bio9, 0.279). This ranking signified that annual precipitation was the most critical factor in LOGT distribution, followed by low winter temperatures. In PC2 (blue arrows), the main factors were the mean temperature of the warmest quarter (bio10, 0.481), the maximum temperature of the warmest month (bio5, 0.481), the mean temperature of the wettest quarter (bio8, 0.356), and temperature seasonality (bio4, 0.277). Therefore, PC2 indicated the impact of temperature on LOGT distribution, emphasizing summer temperatures.

Table 1 presents descriptive statistics of key bioclimatic parameters with a bearing on LOGT distribution areas in China. Temperature seasonality (bio4) exhibited a wide range from 447.1 to 1174.3, with a mean of 878.3 ± 125.9, indicating significant variations in seasonal temperatures across the distribution areas. The maximum temperature of the warmest month (bio5) ranged from 19.0 to 34.6°C, with a mean of 30.6 ± 2.0°C, while the minimum temperature of the coldest month (bio6) varied from −14.6 to 7.4°C, averaging −1.7 ± 4.0°C. Additionally, annual precipitation (bio12) ranged from 433.0 to 1993.0, with a mean of 1113.2 ± 369.4 mm, denoting that precipitation in the distribution area was relatively abundant all year round. However, bio5 and bio10 had a smaller coefficient of variation than the other bioclimatic parameters, with 6.6 and 7.6, respectively. These parameters provided valuable insights into the bioclimatic conditions shaping LOGT distribution in China under the current climate scenario.

**TABLE 1:** Descriptive statistics of the main bioclimatic parameters in the LOGT distribution areas in China.

Bioclimatic variable	Minimum	Maximum	Mean ± SD	Coefficient of variation	95% confidence interval
bio4 Temperature seasonality	447.1	1174.3	878.3 ± 125.9	14.3	868.3 to 888.4
bio5 Max. temperature of the warmest month	19.0	34.6	30.6 ± 2.0	6.6	30.4 to 30.8
bio6 Min. temperature of the coldest month	−14.6	7.4	−1.7 ± 4.0	236.5	−2.0 to −1.4
bio8 Mean temperature of wettest quarter	13.3	27.9	23.6 ± 2.5	10.8	23.4 to 23.8
bio9 Mean temperature of driest quarter	−6.7	17.2	4.7 ± 4.2	89.1	4.4 to 5.0
bio10 Mean temperature of warmest quarter	13.3	28.8	25.3 ± 1.9	7.6	25.2 to 25.5
bio11 Mean temperature of coldest quarter	−6.7	11.9	3.6 ± 3.3	90.8	3.4 to 3.9
bio12 Annual precipitation	433.0	1993.0	1113.2 ± 369.4	33.2	1083.7 to 1142.7

### 3.3 Current potential distribution

**FIGURE 4:**
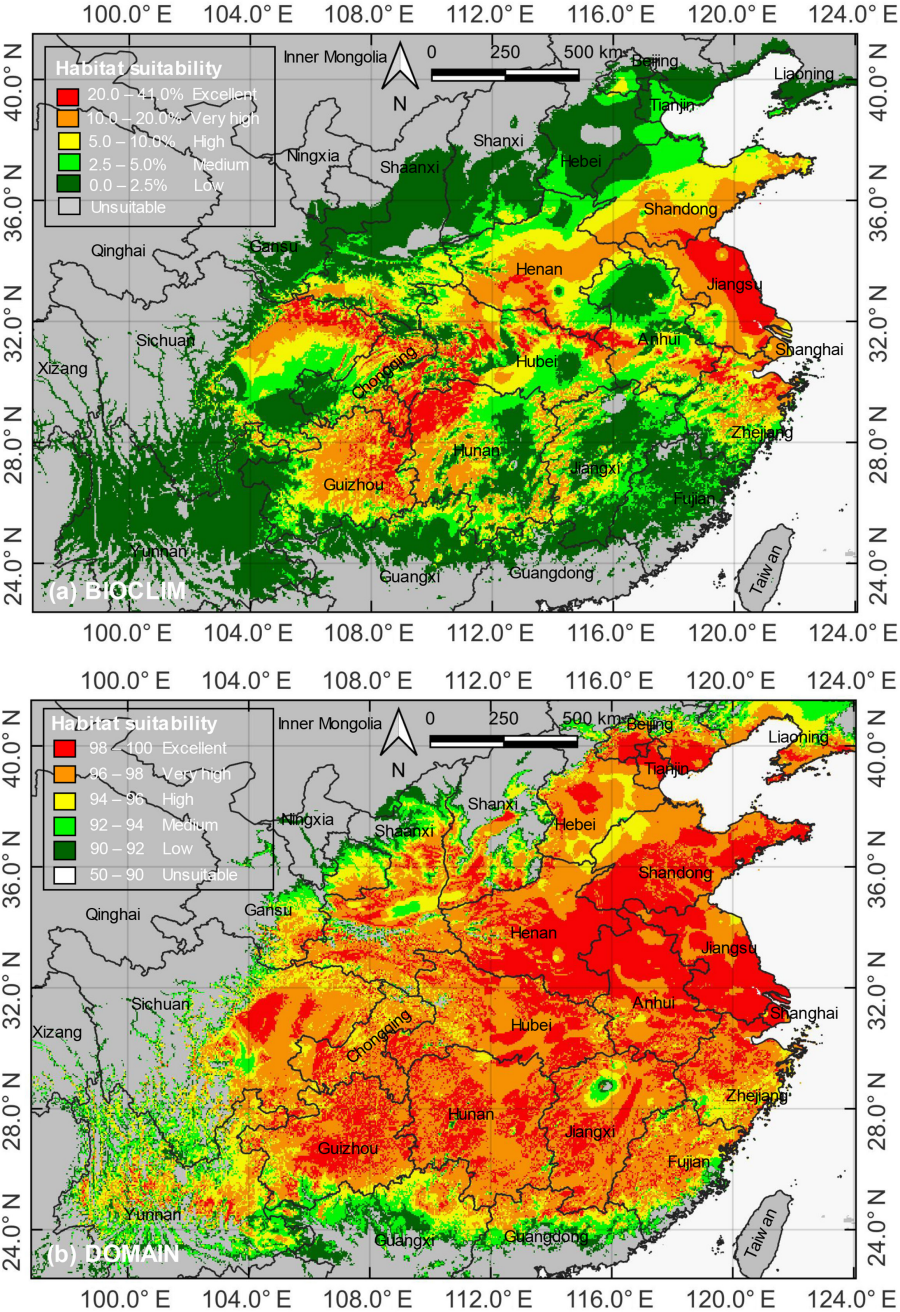
Potentially suitable habitats of LOGTs under the current climate scenario (1970–2000) generated by (a) the BIOCLIM model and (b) the DOMAIN model. The potentially suitable habitats are divided into six categories based on the calculated habitat suitability index, as explained in Section 2.3, and indicated in the legend.

Using DIVA‐GIS version 7.5, we employed the BIOCLIM and DOMAIN models to predict the LOGT potential distribution across China (Figure 4). The results of the BIOCLIM model (Figure 4a) showed that the excellent suitable habitats for LOGTs were scattered in the Jianghuai Plain in eastern China, the Dabie Mountains in central China, and Guizhou, Hunan, Hubei, Chongqing, and Sichuan in western China. Excellent suitable habitats were only sporadically found in other provinces. The results of the DOMAIN model (Figure 4b) indicated that the excellent suitable habitats for LOGTs were more prominent in eastern China (large areas were found in Jiangsu, Anhui, Henan, and Shandong). Guizhou, Chongqing, Sichuan, and Hubei also had some excellent suitability areas. The models' predictions displayed some discrepancies. However, both highlighted Jiangsu, Anhui, Zhejiang, Hubei, Guizhou, Hunan, Chongqing, and Sichuan as core areas with high suitability for LOGTs under the current climate scenario.

By habitat suitability, the total areas for excellent, very high, high, medium, low, and unsuitable categories were 16.5 × 10^4^ km^2^, 44.3 × 10^4^ km^2^, 44.0 × 10^4^ km^2^, 41.4 × 10^4^ km^2^, 116.3 × 10^4^ km^2^, 697.5 × 10^4^ km^2^ for BIOCLIM, and 75.8 × 10^4^ km^2^, 114.1 × 10^4^ km^2^, 43.9 × 10^4^ km^2^, 27.5 × 10^4^ km^2^, 24.8 × 10^4^ km^2^, 673.9 × 10^4^ km^2^ for DOMAIN (Table 2). Compared to other provinces, Jiangsu, Anhui, Henan, and Shandong harbored the largest excellent suitable habitats for LOGTs. DOMAIN identified more areas with excellent, very high, and high suitability levels than BIOCLIM (Table 2). Both models predicted a potential biogeographical range larger than the current one (Figure 1). However, their distinct algorithms yielded noticeably different geographical locations covered by the five suitable habitat categories.

**TABLE 2:** Predicted land areas (×10^4^ km^2^) of potentially suitable habitats for LOGTs under the current and future climate scenarios predicted by BIOCLIM and DOMAIN models. Potentially suitable habitats are classified into six categories.

Suitability category	BIOCLIM			DOMAIN		
	Current	Future	Area change %	Current	Future	Area change %
Excellent	16.5	15.5	−6.1	75.8	71.7	−5.4
Very high	44.3	45.4	2.5	114.1	113.3	−0.7
High	44.0	44.3	0.7	43.9	49.1	11.8
Medium	41.4	43.8	5.8	27.5	32.3	17.5
Low	116.3	119.9	3.1	24.8	27.4	10.5
Unsuitable	697.5	691.1	−0.9	673.9	666.2	−1.1

### 3.4 Predicting changes in suitable habitats

**FIGURE 5:**
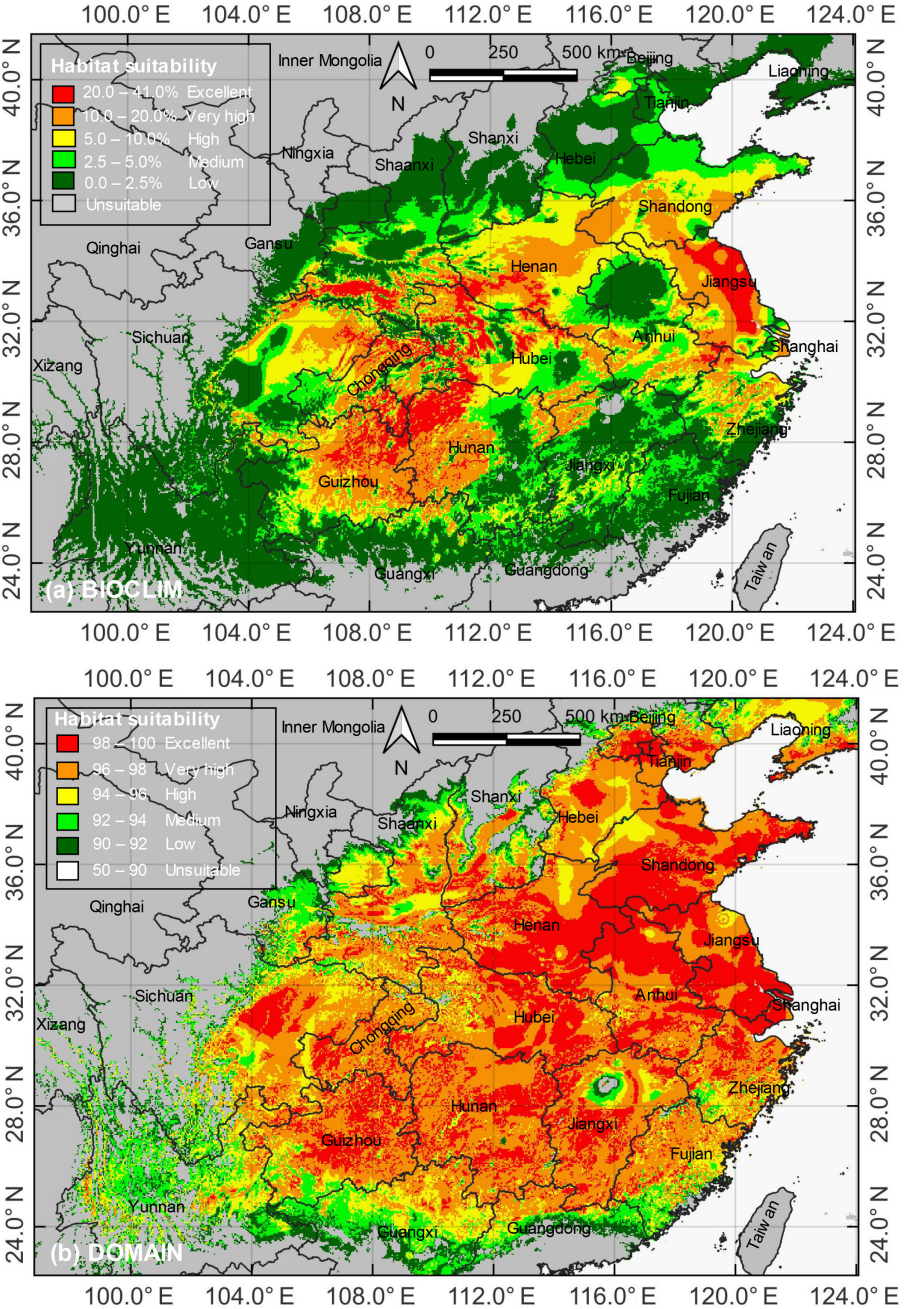
Potentially suitable habitats of LOGTs under the climate change scenario (double CO_2_ concentration) in 2100 generated by (a) the BIOCLIM model and (b) the DOMAIN model. The six categories of potentially suitable habitats are indicated in the legend.

The potentially suitable habitats for LOGTs in the future climatic scenario in 2100 would diverge from the distribution areas under the current climatic scenario (Table 2, Figure 5). The DOMAIN and BIOCLIM models generated different patterns. For BIOCLIM, the excellent suitable habitats decreased by −6.1%, whereas the very high, high, and medium categories increased by 2.5% (45.4 × 10^4^ km^2^), 0.7% (44.3 × 10^4^ km^2^), and 5.8% (43.8 × 10^4^ km^2^). For DOMAIN, the excellent and very high categories decreased by −5.4% (71.7 × 10^4^ km^2^) and −0.7% (113.3 × 10^4^ km^2^), but high and medium categories increased by 11.8% (49.1 × 10^4^ km^2^) and 17.5% (32.3 × 10^4^ km^2^). Therefore, both models predicted the future shrinking of excellent suitable habitats for LOGTs.

**FIGURE 6:**
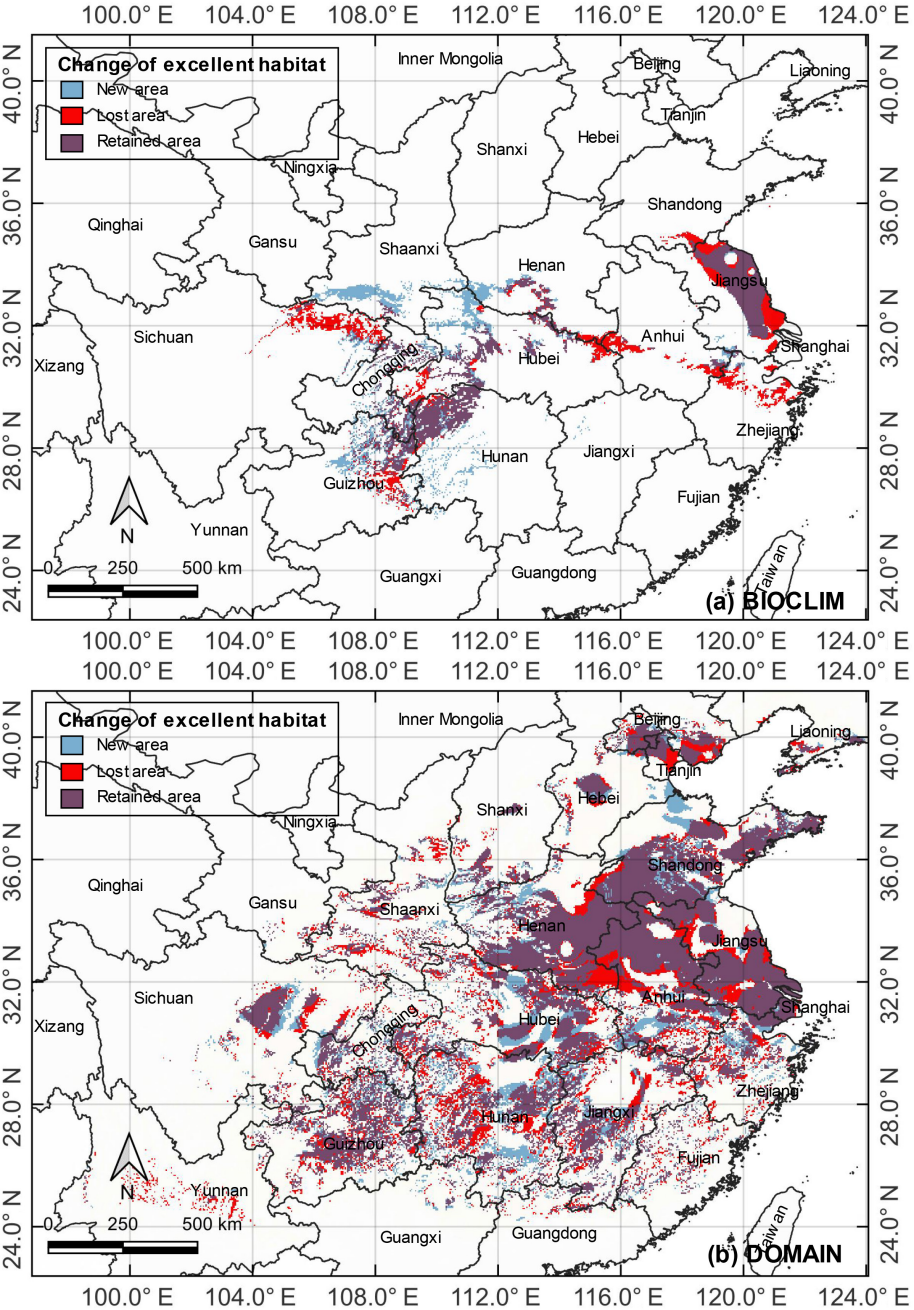
The differences in the distribution of excellent suitable habitats of LOGTs between current and future climate change scenarios (double CO_2_ concentration) generated by (a) the BIOCLIM model and (b) the DOMAIN model.

The excellent suitable habitats of LOGTs under the current and future climate scenarios (Figure 6) were compared by geographical regions. The BIOCLIM results showed significant shrinkage in the Jianghuai Plain, Dabie Mountains, eastern Sichuan, and eastern Guizhou. At the same time, new excellent suitable habitats had emerged in the southern part of Shaanxi and northern Guizhou. Overall, the changing spatial patterns showed signs of northward and westward migration.

The result of the DOMAIN model was somewhat complex. The original excellent suitable habitats were largely retained. Despite losses of excellent suitable habitats, some new ones have appeared in the central and western parts. The pattern of range contraction demonstrated two modes. The larger lost patches were mainly found in central‐eastern China on the peripheral parts of more coterminous distribution areas. Elsewhere, the lost patches were small and scattered.

### 3.5 Model accuracy

Both the current and future cases yielded good average AUC. For BIOCLIM, the AUC values were 0.874 ± 0.0421 and 0.861 ± 0.037, whereas for DOMAIN, they were 0.885 ± 0.026 and 0.892 ± 0.034, respectively. These results signified that the simulations were trustworthy and might be used to investigate how China's LOGT distribution was affected by the current and changing climate.

## 4 DISCUSSION

The distribution area of a species denotes an important spatial characteristic closely related to some pertinent ecological processes, such as species extinction, ecological invasion, and niche amplitude. It is significant for studying species origin, diffusion, and evolution (Bradshaw, 2012; Malliarou et al., 2023). Under the global change background, simulating the potential distribution area of species and analyzing the dominant drivers can provide a scientific basis for effective protection and sustainable utilization of plant resources (Alavi et al., 2019; Girona et al., 2023). With ongoing global change, predicting the potential distribution of species and identifying factors shaping their distribution can strengthen the conservation and management of critical plant resources (Abdelaal et al., 2019; Sheng et al., 2022). Understanding the causes and factors of LOT distribution patterns is particularly important for protecting the unique natural and cultural endowments.

### 4.1 Ginkgo cultivation history and culture in China

Currently, the distribution of naturally spread or artificially introduced *Ginkgo* trees has been recorded in over 20 provinces and regions in China, covering the temperate, warm temperate, and subtropical climatic zones. However, LOGTs are mainly concentrated between 25° and 40° N (Figure 2). The LOGT distribution demonstrated some areas with heavy occurrence, particularly in the border area between Shandong and Jiangsu, southern Anhui, northern Zhejiang, northern Jiangxi, Guangxi, Hubei, Hunan, and Henan, with *Ginkgo* over 100 years old planted in forest patches. Among them, Tancheng in Shandong, Taixing in Jiangsu, Zhuji in Zhejiang, Anlu in Hubei, Shexian in Anhui, and Lingchuan in Guangxi are known as “hometowns” of *Ginkgo* (Liang & Hou, 1990). Tens of thousands of LOGTs in China are over 100 years old (Cao, 2016). Among them, Jiangsu, Hubei, and Zhejiang have very abundant LOGT resources, with 11,858, 9062, and 5000 trees, respectively (Fu et al., 2014; Liu et al., 2019; Sun et al., 2015). Overall, LOGTs in China are mainly distributed in the Yellow River and Yangtze River Basins. The Yellow River Basin includes provinces such as Shanxi, Henan, Shaanxi, and Shandong, whereas the Yangtze River Basin includes provinces such as Hubei, Anhui, and Jiangsu. In addition, some ancient *Ginkgo* trees are widely distributed in China's southwestern regions, such as Yunnan, Guizhou, Sichuan, and Chongqing.

Why are there so many LOGTs in China? From a geological perspective, during the late Tertiary and early Quaternary Glaciation periods, extensive ice covers in the Northern Hemisphere induced drastic environmental changes to endanger *Ginkgo* and many other plant groups (Hsieh, 1992). However, the ice covers in China were not as extensive as those in Europe. The abundance of LOGTs in China can be attributed to the relatively milder impact of glaciers and cold climate in some habitats (Cao, 2016). The relatively subdued cold climate and glaciation in China did not wipe out most plants, leaving some isolated and relatively unaffected pockets in Central and East China, where plants like *Metasequoia glyptostroboides* and *Ginkgo* could survive to become relicts (Liang & Hou, 1990).

China has a long *Ginkgo* cultivation history dating back to over 3600 years ago in the Shang Dynasty (c. 1600–1046 BCE) (Cao, 2016). Subsequently, *Ginkgo* planting increased gradually to expand the range outside the few secluded relict enclaves. By the end of the Han Dynasty (202 BCE–220 CE) and the Three Kingdoms period (220–280 CE), *Ginkgo* was extensively planted in the Yangtze River Basin, with some scattered planting in the Yellow River Basin (Chen & Fan, 2012). The number of cultivated *Ginkgo* in the middle and lower reaches of the Yellow River increased further during the Western Jin (266–317 CE) and Northern and Southern Dynasties (420–589 CE) (Xing et al., 1995). The lingering arboricultural tradition has maintained these regions as *Ginkgo's* main distribution and production areas.

From a religious perspective, Buddhism followers planted and protected large old *Ginkgo* on many temple grounds since about 2000 years ago when the religion was introduced into China. Buddhism's original sacred tree in India was *Ficus religiosa*, a humid tropical species (Sitaramam et al., 2009). However, as the religion spread northward into China and other parts of East Asia, *F. religiosa* could not establish in the northern subtropical and temperate zones in China due to climatic restrictions (Wang et al., 2020). Instead, *Ginkgo* was chosen as a substitute sacred tree among the native tree species pool (Li et al., 2014). Thereafter, Buddhism in China sustained a long tradition of promoting *Ginkgo* cultivation, bringing considerable range expansion, and facilitating the dissemination of the *Ginkgo* spirit and culture (Figure 1a,j). Buddhism provided venues, content, care, and audiences for the dissemination of *Ginkgo* culture (Xu et al., 2023). For example, *Ginkgo* planted in temples can reflect the venues' ancient Buddhism lineage, accompanied by collateral protection of other LOTs species. *Ginkgo*'*s* longevity, robustness, large final size, and dignified tree form complement Buddhism's profound nature‐caring tenet. Buddhism has nurtured a protracted and close connection with *Ginkgo*, which has been revered as sacred since historical times. The highly respected fruits of the *Ginkgo* are considered “sacred,” “immortal,” and “Buddhist” (Chen & Fan, 2013). The *Ginkgo* tree is known as the “Buddhist tree” (Chen & Fan, 2012). Therefore, Buddhism has played an important role in cultivating, protecting, and spreading *Ginkgo* in China (Crane, 2019).

Besides religious connotations, *Ginkgo*'s association with longevity and robust health has led to the deification and worship of some old and robust individual trees (Fu et al., 2014). Chinese people regard *Ginkgo* as auspicious and plant them widely in residential, institutional, and religious grounds to seek shade, cooling, comfort, and blessings (Xing et al., 1995). The deep respect for *Ginkgo* in China is significant for promoting and protecting LOGTs, heightening people's attention to ensure long‐term preservation. People often plant *Ginkgo* to express hope for the prosperity of their descendants and the health and longevity of the elderly in their families (Lu, 2022). Therefore, Chinese traditional Confucian philosophy has actively promoted planting *Ginkgo* and nurturing them into LOGTs. This folk veneration culture has inspired people to plant and protect *Ginkgo* for its symbolic and auspicious significance.

Finally, besides the spiritual and cultural connections, *Ginkgo* offers multiple economic values and vernacular uses. The edible nuts, rich in nutritional value, have been enlisted in various dishes and desserts (Lu, 2022). Additionally, the leaves and seeds are used in traditional Chinese medicine to treat diseases such as cough and asthma (Sun et al., 2015). Furthermore, the hard and durable wood with attractive grains is suitable for making furniture, crafts, and other items (Zhang et al., 2023). Its features suit urban greening purposes, including graceful tree form, large final size, dense foliage, resistance to tree pests and diseases, and good performance. *Ginkgo* is commonly planted in parks, gardens, and roadsides for ornamental and amenity purposes. From the secular perspective, its varied economic worth and practical uses have encouraged cultivation in cities and the countryside.

### 4.2 Current LOGT distribution and key environmental drivers

Based on the current climate conditions, the LOGTs (Figures 2 and 4) show a distribution pattern generally confined to the north of the subtropical Lingnan Mountains and south of the cool‐temperate Liaodong Peninsula. Within this broad latitudinal belt, the LOGTs extend from the southeastern coastal areas' core range to the inland southwest region. Their occurrence gradually decreases toward the southern limit of the current range at about 30° N.

PCA results indicated that the annual precipitation (bio12) significantly influenced LOGT distribution (Figure 3). The notable concentration of LOGTs in the coastal provinces of eastern China (Figure 2) coincides with the Pacific monsoon, bringing abundant precipitation to this region (Gao et al., 2014). Moving westward, precipitation gradually decreases, reducing the LOGT population compared to the eastern regions. The ample precipitation in China's southeastern and southwestern regions ensures the establishment and persistence of LOGTs (Table 2). An innate biological trait of *Ginkgo*, sperms with flagella that demand water for mobility, means sufficient rainfall is essential for successful reproduction (Shi, 1992).

Temperature is the other key factor limiting the distribution of LOGTs in China. Among the first four factors associated with PC1 and PC2, only one is associated with moisture (bio12 annual precipitation), and the remaining factors are related to temperature (Figure 3). Plants must conduct photosynthesis and respiration within a certain temperature range, requiring optimal temperature thresholds for normal growth (Criddle et al., 1997). *Ginkgo* temperature tolerance experiments demonstrated that after 4 h of treatment at 43°C, the leaves wilted severely. The experimental results indicated a sudden decrease in the defense activities of SOD and POD (superoxide dismutase and peroxidase) enzymatic antioxidants. After 16 h of treatment at 45°C, the *Ginkgo* plant died. Conversely, after 4 h of treatment at subzero temperatures, SOD and POD activities in the detached Ginkgo leaves continued to increase. The relative conductivity of the leaves (21.34%) was much lower than the lethal relative conductivity standard (50%) (Gu et al., 2005). Therefore, *Ginkgo* has poor heat resistance but some cold resistance (Shi, 1992). The lack of LOGTs in the warmest south subtropical zone and extensive spread from the north subtropical to temperate zones of China reflects the species' temperature tolerance limit.

The flowering phenology of LOGTs at the southern end of their distribution is highly correlated with the average temperature in February of the same year. The region's annual low temperatures usually occur in mid to late January (Ding et al., 2022). After experiencing this timely low‐temperature vernalization treatment, Ginkgo's flower bud differentiation and growth must occur at relatively high temperatures (Feng et al., 1999). Experimental evidence has shown that *Ginkgo* buds sprout at temperatures above 8°C, and organ growth begins above 12°C (Liang, 1993). The gradually increasing ambient temperature after the cold vernalization is conducive to the accumulation of organic matter, thereby promoting *Ginkgo* flowering (García et al., 2000). Therefore, the lack of vernalization opportunities in the south tropical zone has restricted *Ginkgo's* southward spread.

Overall, the geographical distribution of *Ginkgo* is closely related to its inherent physiological characteristics. The species prefers abundant precipitation and is unsuitable for areas with extremely high or low temperatures.

### 4.3 Spatial distribution under future climate change scenarios

The combined predictions of the BIOCLIM and DOMAIN models paint a stark picture of LOGT's future in China under a doubled CO_2_ scenario. Our findings resonate with previous studies underscoring *Ginkgo's* limited tolerance to climatic fluctuations (Feng et al., 2023; Wang et al., 2023). In particular, the projected decrease in excellent suitable habitats signifies a significant threat to the long‐term survival of the living fossils. This decline, coupled with the anticipated northward and westward shift of optimal habitats (Feng et al., 2023), suggests a gradual retreat toward higher latitudes, mirroring similar trends observed in other plant species (Abdelaal et al., 2019). This northward shift takes advantage of the emergence of new suitable habitats. However, their fragmented nature, with reference to individual patch size and interpatch distance, poses critical challenges. The biogeographical barriers of extensive farmlands and settlements would present migration challenges. The likely consequences include reduced connectivity and gene flow between isolated subpopulations, potentially compromising population resilience and adaptation (Cao, 2007). Additionally, existing populations at the southern edge of their current range will face increasingly acute stresses as climatic conditions shift beyond their optimal tolerances, impacting growth, reproduction, and survival.

Under future climate change scenarios, many plants are migrating poleward or to higher altitudes. First, warming climates modify the climatic conditions of native ecosystems, rendering some plant habitats unsuitable. Adapting to the new climate, plants abandon or dodge unsuitable areas by migrating to suitable habitats. Second, climate change affects plant growth by shortening the original growing season or contracting the ecological niche. Additionally, climate change may lead to the collapse or degradation of some ecosystems, forcing plants to seek new habitats. As *Ginkgo* has a certain cold resistance (Gu et al., 2005), LOGTs will likely encounter deteriorating environmental conditions and range contraction at middle and low altitudes under future warming climates.

Due to cultural imperative, *Ginkgo* has always attracted much attention in China. Previous studies employing different modeling approaches offer insights into the potential mode and magnitude of possible habitat changes. However, the area of *Ginkgo's* suitable habitats showed contrasting results in various studies: increasing (Zhang et al., 2023) or decreasing (Feng et al., 2023; Guo et al., 2019; Wang et al., 2023). The anomaly may be related to the selection of models and specific research objects (Araújo & Guisan, 2006). First, SDMs using different algorithms and parameters may have different sensitivities and responses to climate change, leading to different predictive results (Wisz et al., 2008). For example, some models are more sensitive to temperature changes, whereas others are more sensitive to precipitation changes (Brun et al., 2020). Second, the input data set of SDM models includes species' current distribution and climate data (Arenas‐Castro et al., 2022). The data collection method and accuracy of the species' current distribution information have an important bearing on a model's prediction. In addition, the source and accuracy of climate data will affect the modeling outcome (Kadmon et al., 2003).

Nevertheless, the impact of climate change on LOGT distribution is inevitable. The reliable projections call for preventive and ameliorative measures to reduce the impacts on important biological resources. Based on this study's findings, we recommend implementing targeted protection measures in the retained, new, and lost areas. The retained areas, with better suitable habitats for the distribution of LOGTs, offer more assured refuges under future climate change scenarios and main locations for experimental research. For the new areas, *Ginkgo* planting can be increased to provide sufficient backup resources for cultivating the species throughout the potentially suitable habitats. In the lost areas, it is important to conduct resource surveys and dynamic monitoring of LOGTs to improve the growth‐site conditions and adaptability of LOGTs to climate change. If feasible, some important LOGTs can be relocated to locations within suitable habitats.

### 4.4 Implications for conservation

The implications of our findings extend beyond the fate of LOGTs per se. As these living fossils act as ecological indicators, their decline affords a glimpse into the broader impacts of climate change on biodiversity and ecosystem functioning. In this context, our work underscores the need for proactive and preventive conservation strategies to safeguard these iconic trees, the unique ecosystems they support, and the cultural services they provide.

However, the current survival status of the ancient *Ginkgo* trees cannot be rated as optimistic. They face several crucial challenges: (1) Natural factors. Due to old age, physiological decline, and reduced stress resistance, LOGTs are susceptible to natural degradations such as pest and disease intrusion, dieback, breakage, and collapse, hindering growth and driving them toward decline and death (Lin et al., 2022). Climate change has also imposed adverse effects on the distribution and survival of LOGTs, such as elevated temperature, reduced precipitation, and intensified drought (Lindenmayer et al., 2012; Lloret & Batllori, 2021). (2) Human factors. Due to their historical and cultural value, LOGTs are commonly cherished by people. However, this affinity has brought undesirable illegal logging, theft, and trafficking, leading to a sharp decrease in their numbers and may threaten species survival (Müllerová et al., 2014). Additionally, urbanization involving land development, infrastructure construction, and environmental pollution has jeopardized the survival of ancient trees by damaging their soil conditions and root systems, reducing their light exposure, and polluting their ambient air and soil (Chi et al., 2020). (3) Management factors. Deficiencies in LOGT protection and management have lingered on a broad front (Lindenmayer & Laurance, 2017; Wu et al., 2020). They involve the lack of or incomplete surveys, improper or absence of monitoring, substandard or harmful maintenance, inadequate protection by laws, insufficient funding, and unprofessional personnel. These chronic problems affect the health and safety of LOGTs, increasing their survival risks.

We suggest specific mitigating and preventive measures to bring improvements: (1) Establishing a coordinated mechanism for LOGT protection and management. It calls for resolving conflicts and detachment among the cognate realms of cultural relicts, environment, and construction. This goal could be achieved through communication and cooperation among principal stakeholders, such as cultural heritage, forestry, nature conservation, urban and rural planning, and landscape management (Yao et al., 2024). (2) Using modern scientific knowledge and techniques. It aims to accurately identify, analyze, and predict the LOGT resource status, distribution characteristics, growth environment, and potential threats. The outputs can provide a scientific and effective basis for their protection and management (Dudkiewicz & Durlak, 2021). Additionally, advanced arboricultural techniques could be adopted to maintain the health and vitality of LOGTs, such as watering, drainage, fertilization, pest control, crown pruning, environmental improvement, and preventive protection. (3) Integrating local folklore and culture. It can interpret and promote the multiple values of LOGTs through education and publicity. Traditional and new media can be enlisted to strengthen the citizen science approach to disseminate the historical, cultural, ecological, and scientific values of LOGTs and raise public awareness and participation in their protection (Blicharska & Mikusiński, 2014; Huang et al., 2020). (4) Collecting, preserving, and utilizing high‐quality germplasm resources of LOGTs. This fundamentally pertinent measure can preserve the irreplaceable genetic composition and facilitate their reproduction, nursery production, and planting programs (Chi et al., 2020; Zhou et al., 2020) to sustain genetic diversity and long‐term species and tree survival.

## 5 CONCLUSION

The present geographic distribution pattern of LOGTs in China covers a broad latitudinal range across northern subtropical and temperate zones in central and eastern China, with a concentration in low elevations and coastal regions. The Principal Component Analysis (PCA) reveals that annual precipitation and temperatures are the primary factors influencing the distribution pattern. Statistical analysis of key bioclimatic parameters highlighted substantial variations in seasonal temperature fluctuations across the distribution areas.

The BIOCLIM and DOMAIN models predicted excellent suitable habitats, primarily in eastern and southwestern China, with some discrepancies in their predictions. Notably, provinces in the eastern and southwestern regions with sufficient precipitation and freedom from excessively low temperatures in winter are identified as core areas with high suitability for LOGTs under the current climate scenario. A good proportion of excellent habitats will be lost, and the remaining ones will suffer from fragmentation.

The future climate change scenario of doubled CO_2_ concentration anticipated tree decline and habitat losses, especially prominent at the southern edge of the range. A proportion of the present range in the core distribution areas will be retained. Some new suitable habitats will emerge to allow range expansion to compensate partly for the losses. Nevertheless, their small patch size and large interpatch distance will induce isolation and fragmentation of the subpopulations, with implications on restricted gene flow.

In sum, the comprehensive analysis of spatial and altitudinal distribution patterns, coupled with the identification of key bioclimatic growth factors, provides valuable insights for understanding and managing LOGTs in China under climate change scenarios. The findings contribute to this unique plant species' effective conservation and sustainable management amidst ongoing global environmental changes.

## AUTHOR CONTRIBUTIONS

**Chunping Xie:** Data curation (equal); methodology (equal); writing – original draft (equal). **Chang Liu:** Data curation (equal); investigation (equal); visualization (equal). **Houhe Wang:** Data curation (equal); investigation (equal); visualization (equal). **Dawei Liu:** Formal analysis (equal); investigation (equal). **Chi Yung Jim:** Formal analysis (equal); software (equal); validation (equal); writing – original draft (equal).

## FUNDING INFORMATION

This research was funded by the National Natural Science Foundation of China (grant number: 32360417), the Natural Science Foundation of Hainan Province (grant number: 423MS061), and the Education Department of Hainan Province (project number: Hnky2023ZD‐17).

## CONFLICT OF INTEREST STATEMENT

The authors declare that they have no known competing financial interests or personal relationships that could have appeared to influence the work reported in this paper.

## Data Availability

Geographical data in this manuscript are available on Science Data Bank https://www.scidb.cn/s/UbIr2y.
